# Risk factors associated with hemodialysis central venous catheter malfunction; a retrospective analysis of a randomized controlled trial

**DOI:** 10.1186/2054-3581-1-15

**Published:** 2014-07-08

**Authors:** David R Ward, Louise M Moist, Jennifer M MacRae, Nairne Scott-Douglas, Jianguo Zhang, Marcello Tonelli, Charmaine E Lok, Steven D Soroka, Brenda R Hemmelgarn

**Affiliations:** Department of Medicine, University of Calgary, 1403 29th St NW, Calgary, AB T2N 2 T9 Canada; London Health Sciences Center, University of Western Ontario, London, Ontario Canada; Department of Medicine, University of Alberta, Edmonton, Alberta Canada; University Health Network-Toronto General Hospital, University of Toronto, Toronto, Ontario Canada; Department of Medicine, Dalhousie University, Halifax, Nova Scotia Canada

**Keywords:** Catheters, Equipment, Malfunction, Hemodialysis, Renal dialysis, Risk factors

## Abstract

**Background:**

We previously reported a reduction in central venous catheter (CVC) malfunction when using once-weekly recombinant tissue-plasminogen activator (rt-PA) as a locking solution, compared with thrice-weekly heparin.

**Objectives:**

To identify risk factors for CVC malfunction to inform a targeted strategy for rt-PA use.

**Design:**

Retrospective analysis.

**Setting:**

Canadian hemodialysis (HD) units.

**Patients:**

Adults with newly placed tunnelled upper venous system CVCs randomized to a locking solution of rt-PA(1 mg/mL) mid-week and heparin (5000 u/ml) on the other HD sessions, or thrice-weekly heparin (5000 u/ml).

**Measurements:**

CVC malfunction (the primary outcome) was defined as: peak blood flow less than 200 mL/min for thirty minutes during a HD session; mean blood flow less than 250 mL/min for two consecutive HD sessions; inability to initiate HD.

**Methods:**

Cox regression was used to determine the association between patient demographics, HD session CVC-related variables and the outcome of CVC malfunction.

**Results:**

Patient age (62.4 vs 65.4 yr), proportion female sex (35.6 vs 48.4%), and proportion with first catheter ever (60.7 vs 61.3%) were similar between patients with and without CVC malfunction. After multivariate analysis, risk factors for CVC malfunction were mean blood processed < 65 L when compared with ≥ 85 L in the prior 6 HD sessions (HR 4.36; 95% CI, 1.59 to 11.95), and mean blood flow < 300 mL/min, or 300 – 324 mL/min in the prior 6 HD sessions (HR 7.65; 95% CI, 2.78 to 21.01, and HR 5.52; 95% CI, 2.00 to 15.23, respectively) when compared to ≥ 350 mL/min.

**Limitations:**

This pre-specified post-hoc analysis used a definition of CVC malfunction that included blood flow, which may result in an overestimate of the effect size. Generalizability of results to HD units where trisodium citrate locking solution is used may also be limited.

**Conclusions:**

HD session characteristics including mean blood processed and mean blood flow were associated with CVC malfunction, while patient characteristics were not. Whether targeting these patients at greater risk of CVC malfunction with rt-PA as a locking solution improves CVC longevity remains to be determined.

## Abrégé

**Contexte:**

Nous avons précédemment fait rapport d’une réduction du dysfonctionnement du cathéter veineux central (CVC) lors de l’utilisation hebdomadaire de l’activateur tissulaire du plasminogène obtenu par génie génétique (rt-PA) comme solution verrou, plutôt que l’administration d’héparine trihebdomadaire.

**Objectifs:**

Déterminer les facteurs de risques de dysfonctionnement du CVC afin d’indiquer une stratégie visée quant à l’utilisation du rt-PA.

**Type d’étude:**

Analyse rétrospective.

**Contexte:**

Les services canadiens d’hémodialyse (HD).

**Participants:**

Adultes à qui on a nouvellement installé un CVC tunnellisé intravasculaire dans le système veineux supérieur, et qui ont reçu soit une solution verrou de rt-PA (1 mg/ml) en milieu de semaine et de l’héparine (5 000 u/ml) lors des autres séances d’HD, soit de l’héparine trois fois par semaine (5 000 u/ml).

**Mesures:**

On a défini le dysfonctionnement du CVC (résultat primaire) comme étant : un débit sanguin de pointe inférieur à 200 mL/min durant trente minutes, au cours d’une séance d’HD; un débit sanguin moyen inférieur à 250 mL/min lors de deux séances d’HD consécutives; l’impossibilité d’entamer l’HD.

**Méthodes:**

On a eu recours au modèle de régression de Cox pour déterminer l’association entre les données démographiques des participants, les variables relatives au CVC lors des séances d’HD et le résultat d’un dysfonctionnement du CVC.

**Résultats:**

L’âge des participants (62,4 c. 65,4 ans), la proportion des participants de sexe féminin (35,6% c. 48,4%), et la proportion de ceux à qui on a installé un cathéter pour la première fois (60,7% c. 61,3%) étaient similaires entre les patients qui ont subi un dysfonctionnement du CVC et ceux qui n’en ont pas subi. L’analyse multifactorielle révèle que les facteurs de risque liés au dysfonctionnement du CVC sont un traitement moyen du sang < 65 L, comparativement à ≥ 85 L au cours des 6 séances d’HD précédentes (HR 4,36; 95% CI, 1,59 à 11,95), et un débit sanguin moyen < 300 mL/min, ou 300 – 324 mL/min lors des 6 séances d’HD précédentes (HR 7,65; 95% CI, 2,78 à 21,01, et HR 5,52; 95% CI, 2,00 à 15,23, respectivement), comparativement à ≥ 350 mL/min.

**Limites de l’étude:**

Cette analyse prédéterminée et post-hoc reposait sur une définition du dysfonctionnement du CVC qui comprenait le débit sanguin, ce qui pourrait résulter en une surestimation de l’ampleur de l’effet réel. La validité externe des résultats pour les services d’HD qui utilisent le citrate trisodique comme solution verrou pourrait aussi être limitée.

**Conclusions:**

Les caractéristiques des séances d’HD comprenant le traitement moyen du sang et le débit sanguin moyen ont été associées au dysfonctionnement du CVC, alors que les caractéristiques des participants ne l’ont pas été. Il reste à déterminer si le fait de cibler les patients courant un risque accru de dysfonctionnement du CVC avec rt-PA comme solution verrou améliore la longévité du CVC.

## What was known before

Central venous catheters (CVC) are commonly used to establish access in adult North American hemodialysis patients. CVC malfunction is a common clinically encountered complication of the use of tunnelled CVC. Previous authors have identified patient and CVC characteristics associated with malfunction.

## What this adds

In this pre-specified, post-hoc analysis of data from a randomized controlled study we found that patient characteristics were not associated with risk of CVC malfunction after adjusting for hemodialysis session characteristics. Further research is required to predict CVC malfunction and strategies for prevention.

## Background

Current guidelines recommend hemodialysis (HD) vascular access via an arterio-venous fistula. However, central venous catheters (CVC) are used by approximately 70% of incident HD patients in North America [1–5]. Complications associated with CVC use include thrombosis and decreased dialysis adequacy due to catheter malfunction [6–8]. These complications are associated with increased healthcare resource utilization; up to 50% of all tunnelled CVC’s fail within one year [9–12].

CVC locking solutions are used in the interdialytic period to decrease the risk of complications; the optimal strategy to minimize CVC complications remains to be determined [13–16]. We previously reported that using once-weekly rt-PA as a locking solution leads to a two-fold reduction in CVC malfunction, compared with thrice-weekly heparin [17]. However, given the potential costs associated with this strategy, its use and uptake may be limited. Identifying patients at greatest risk of CVC malfunction would permit targeted use of this strategy. Using data from the prior randomized trial [17] we sought to determine the association between patient characteristics and CVC-related variables during a dialysis session and the outcome of CVC malfunction.

## Methods

The subjects and design of the original trial have been described previously [18]. In summary, adult HD patients with newly inserted tunnelled, upper venous system HD catheters were eligible. Subjects were excluded if they were on systemic anticoagulation, undergoing current antibiotic use for catheter related bacteremia, the CVC was inserted via guidewire exchange, or they had risk factors for increased bleeding. If patients with catheter-related bacteremia completed antibiotic treatment, and remained bacteremia free for three hemodialysis sessions, they were eligible. CVC reversal was permitted for up to three consecutive hemodialysis sessions after which the study protocol mandated the CVC run in the non-reversed orientation and the patient was then eligible for the primary outcome.

The study was conducted in adherence with the Declaration of Helsinky, approved by the research ethics board of each institution, and patients provided written, informed consent. Patients were randomized to either thrice-weekly catheter locking solution with heparin (5000 u/mL, full luminal volume), or substitution of the mid-week heparin with recombinant tissue plasminogen activator (rt-PA) (1 mg in 1 mL with saline to full luminal volume) and standard heparin catheter locking solution following the other dialysis sessions. All other HD care was per site-specific protocols.

CVC malfunction (the primary outcome) was defined as: peak blood flow less than 200 mL/min for thirty minutes during a HD session; mean blood flow less than 250 mL/min for two consecutive HD sessions; inability to initiate HD [19]. Each patient was followed-up for six months. Patients were censored at the first of: study end, removal of the CVC, initiation of alternative renal replacement therapy, or transfer to a centre not involved in the trial.

### Candidate risk factors for CVC malfunction

Potential risk factors included patient characteristics (age, gender, race, duration of dialysis, cause of ESRD), baseline blood work (hemoglobin, platelets, albumin), comorbidity (prior myocardial infarction, congestive heart failure, diabetes, pulmonary embolism, gastrointestinal bleed, deep venous thrombosis, hypertension, cancer, current smoker), type of treatment (heparin-only or rt-PA), and CVC characteristics (any previous CVC, number of prior CVCs, insertion site, indication). We also considered HD-specific variables (mean blood processed, mean blood flow, mean arterial pressure, mean venous pressure) in the immediate HD session, as well as the 6 HD sessions, prior to CVC malfunction (for those with a primary outcome) or the same time period prior to censoring (for those without a primary outcome). We adjusted for CVC reversal given its potential role as a confounder, and assessed its role as a risk factor in a univariate analysis.

### Statistical analysis

Cox proportional hazards regression analyses for time to catheter malfunction (for those with an event) or censoring (for those without an event) were performed, initially with all candidate risk factors for CVC malfunction assessed in a univariate fashion, followed by multivariate analyses with adjustment for type of treatment, age, gender, first dialysis catheter ever, diabetes, history of rt-PA use with a prior CVC and CVC reversal. All statistical tests were two-sided, and reported P values were considered significant if less than 0.05. All analyses were performed using STATA 11.2 (Stata Corporation, College Station, Texas).

## Results

We included all 225 patients from the original randomized trial in this study. Baseline characteristics were similar between patients with CVC malfunction and patients with no CVC malfunction (Table 1). One hundred sixty-three patients (72.4%) had no CVC malfunction and sixty-two (27.6%) patients had malfunction. The median duration of follow-up was 162 days for patients with no malfunction and 48.5 days for patients with CVC malfunction.

**Table 1:** **Baseline characteristics**

Patient characteristics	Overall (N = 225)	No CVC malfunction (N = 163)	CVC malfunction (N = 62)
**Treatment**			
Rt-PA Group	110 (48.9%)	88 (54.0%)	22 (35.5%)
Heparin only Group	115 (51.1%)	75 (46.0%)	40 (64.5%)
**Age -yr, mean ± SD**	63.2 (15.9)	62.4 (16.6)	65.4 (14.0)
**Female sex – no. (%)**	88 (39.1%)	58 (35.6%)	30 (48.4%)
**Duration of dialysis –yr, median (IQR)**	1.0 (0.0 - 6.0)	1.0 (0.0 - 6.0)	0.0 (0.0 - 1.0)
**Central venous catheter Indication - no. (%):**			
Starting dialysis without peripheral access	118 (52.4%)	84 (51.5%)	34 (54.8%)
Failure of/awaiting peripheral access	48 (21.3%)	32 (19.6%)	16 (25.8%)
Catheter related infection	14 (6.22%)	11 (6.75%)	3 (4.84%)
**First dialysis catheter ever - no. (%)**	137 (60.9%)	99 (60.7%)	38 (61.3%)
**Cause of end-stage renal disease:**			
Diabetic nephropathy	71 (31.6%)	48 (29.4%)	23 (37.1%)
Glomerulonephritis	17 (7.56%)	12 (7.36%)	5 (8.06%)
Hypertension or vascular disease	42 (18.7%)	35 (21.5%)	7 (11.3%)
**Coexisting or prior illness - no. (%):**			
Diabetes mellitus	124 (55.1%)	86 (52.8%)	38 (61.3%)
Hypertension	206 (91.6%)	150 (92.0%)	56 (90.3%)
Ischemic heart disease	47 (20.9%)	34 (20.9%)	13 (21.0%)
**Hemoglobin - g/liter, mean ± SD**	106 (15.7)	106 (15.8)	106 (15.4)
**Albumin – g/liter, mean ± SD**	32.1 (6.5)	32.1 (6.7)	32.0 (5.9)
**Platelet count - x10-9/litre, median (IQR)**	230 (185–293)	233 (185–298)	220 (183–283)

IQR: interquartile range; SD: standard deviation.

The study period included 24,085 CVC days; 3,048 in the patients with CVC malfunction and 21,037 in the patients with no CVC malfunction. Of the 62 patients with CVC malfunction, 40 (64.5%) were treated with heparin only. Of the 163 patients with no CVC malfunction, 75 (46.0%) were treated with heparin only. Univariate analysis found a significant, increased risk of CVC malfunction when HD lines were reversed at the session immediately prior to malfunction (hazard ratio [HR] 11.2, 95% CI 6.35-19.7, p < 0.001), as well as at least once in the six sessions prior to malfunction (HR 9.50, 95% CI 4.49 – 20.10, p < 0.001). Within the 6 antecedent HD sessions to the primary outcome, risk increased with an increasing number of sessions (to a maximum of three as per protocol) with CVC reversal (HR 1.72; 95% CI 1.51 – 1.96, p < 0.001), and in patients with a history of rt-PA use for catheter malfunction (HR 1.89 95% CI 1.04-3.46 p = 0.038). The risk of CVC malfunction decreased as blood processed or mean blood flow increased (p < 0.001). A trend to increased risk was found for female patients (HR 1.57 95% CI 0.95-2.59 p = 0.075). Age, diabetes, dialysis duration, first CVC status, hemoglobin, and number of CVC in the prior year were not significantly associated with malfunction (Table 2).

**Table 2:** **Univariate analysis of potential risk factors for CVC malfunction**

Variable	Hazard ratio (95% CI)	P-value
Treatment group		0.02
Heparin-only	1.91 (1.13, 3.22)	
rt-PA	Ref.	
Age	1.01 (0.99, 1.03)	0.275
Duration of dialysis	0.59 (0.26, 1.38)	0.224
Number of catheters in prior year	1.20 (0.92, 1.57)	0.172
Hemoglobin	1.00 (0.98, 1.02)	0.950
Albumin	1.00 (0.96, 1.04)	0.927
Platelets	1.00 (1.00, 1.00)	0.509
Gender		0.073
Male	Ref.	
Female	1.57 (0.95, 2.59)	
First dialysis catheter ever		0.878
No	Ref.	
Yes	1.04 (0.62, 1.74)	
Diabetes		0.352
No	Ref.	
Yes	1.27 (0.76, 2.12)	
History of rt-PA use for catheter malfunction		0.082
No	Ref.	
Yes	1.89 (1.04, 3.46)	
CVC reversed at the session prior to the event or censoring:		<0.001
No	Ref.	
Yes	11.2 (6.35, 19.7)	
> = 1 CVC reversed in the 6 sessions prior to the event or censoring:		<0.001
No	Ref.	
Yes	9.50 (4.52, 20.0)	
Number of HD sessions with CVC reversed in the 6 sessions prior to the event or censoring	1.71 (1.51, 1.93)	<0.0001
Mean blood processed in the prior 6 runs (L) < 65	5.26 (2.00 – 13.87)	<0.001
65 - 74	3.23 (1.22 – 8.58)	
75 - 84	1.50 (0.51 – 4.41)	
85+	Reference	
Mean blood flow in the prior 6 runs (mL/min)		<0.001
< 300	8.45 (3.20 –22.34)	
300 - 324	6.80 (2.49 – 18.56)	
325 - 349	2.77 (0.90 – 8.49)	
350+	Reference	

In a multivariable analysis the only variables significantly associated with CVC malfunction were HD session characteristics. After adjusting for baseline characteristics (age, gender, diabetes, first dialysis catheter ever), treatment allocation, history of rt-PA use, and CVC line reversal, patients with mean blood processed < 65 L had significantly higher risk of CVC malfunction when compared with patients with mean blood processed ≥ 85 L in the prior 6 HD sessions (HR 4.36; 95% CI, 1.59 to 11.95). When compared with patients with mean blood flow (averaged over the antecedent six sessions) ≥ 350 mL/min, those with mean blood flow < 300 mL/min and those with mean blood flow 300 to 324 mL/min had significantly higher risk of CVC malfunction (HR 7.65; 95% CI, 2.78 to 21.01 for the former comparison, and HR 5.52; 95% CI, 2.00 to 15.23 for the latter comparison) (Table 3).

**Table 3:** **Multivariable analysis of predictors of catheter malfunction**

Predictor variable	Adjusted* HR (95% CI)
Mean blood processed in the prior 6 runs (L)	
< 65	4.36 (1.59-11.95)
65 - 74	2.65 (0.98-7.12)
75 - 84	1.31 (0.44-3.95)
85+	Reference
Mean blood flow in the prior 6 runs (mL/min)	
< 300	7.65 (2.78-21.01)
300 - 324	5.52 (2.00-15.23)
325 - 349	2.37 (0.76-7.45)
350+	Reference

*Adjusted for age (years), gender, treatment allocation, first dialysis catheter ever, diabetes, history of rt-PA use with prior CVC, CVC reversed in prior run, CVC reversed at least once in the prior 6 runs, and number of runs with CVC reversed in the prior 6 runs.

## Discussion

In this pre-specified post-hoc analysis of data from a randomized controlled study we quantified the risk for CVC malfunction conferred by routinely observed and monitored patient and HD session characteristics. We found a significant increased risk when a reduction in litres of blood processed and mean blood flow over a two-week period occurred, however there was no association between baseline patient characteristics and risk of CVC malfunction.

CVC malfunction is the most common cause for CVC removal [20–22]. However, few studies have identified and quantified risk factors or predictors of CVC malfunction. Previous studies of patients with tunnelled CVCs reported that diabetic status and prior CVC exposure were associated with malfunction [12, 23]. However, data lacking on malfunction definition, locking solution, and the inclusion of infections and non-infectious criteria for CVC removal make extrapolation of these results difficult. While CVC location has been identified as a risk factor for malfunction, this study examined non-tunnelled catheters only [24]. An observational cohort study of 3,364 incident and prevalent HD patients with a CVC in the United States reported the risk of CVC malfunction (blood flow <300 mL/min) was lower for males and black race (compared with white) and higher if CVC was not the first access modality [25]. Male patients in our study were also at decreased risk but, possibly due to small numbers, we saw no significant effect of first CVC ever or ethnicity.

We analysed six sessions (a two week period) as we felt it would encompass a reasonable timeframe for clinicians to review dialysis characteristics in real-world practice; potentially guiding decision-making to pre-empt malfunction. In univariate analysis we found the strongest risk factor for CVC malfunction was CVC reversal in the prior HD session, or any of the six sessions prior to malfunction. However, our study protocol limited the number of consecutive sessions that could be performed in the reversed position to three, therefore this variable was included as a potential confounder in the adjusted analysis rather than an independent predictor given its potential relationship with the outcome. KDIGO Guidelines [19] suggest that CVC reversal be a temporary solution to managing malfunction, and authors have found that one third of patients may routinely undergo dialysis with their catheters in the reversed position [26]. This prevalence may be influenced by studies questioning the impact of CVC line reversal or blood flow on dialysis adequacy. Carson et al. noted CVC reversal improved clearance, despite increased recirculation, if the blood flow through the CVC concurrently increased to an average 315 mL/min [27] and Moist et al. reported decreased blood flow through reversed CVCs did not significantly affect online urea clearance if blood flow was greater than 250 mL/min [28]. Our results corroborate these findings as adjusted subgroup analysis by blood flow or blood processed quartile found a statistically significant increased risk when blood flows were less than 325 mL/min or total blood processed was less than 65 L per session in the preceding six sessions to a malfunction event.

We sought to pragmatically identify HD session characteristics that may signal an increased risk of CVC malfunction, and warrant consideration for early intervention. Decreased blood flow below 325 mL/min or total blood processed less than 65 L per session averaged over six sessions may therefore signal early or mild CVC malfunction. Mechanistically, it is plausible that a developing CVC thrombus or fibrin sheath, partially obstructing the CVC, and will decrease the average blood flow and blood processed. Interventions such as thrombolytics, catheter stripping, or catheter exchange may be required to improve CVC function, although these interventions are associated with costs, and long term catheter patency rates are suboptimal irrespective of intervention chosen [29, 30]. CVC reversal, decreased blood flow, or decreased blood processed may herald the future onset of severe malfunction (as defined by blood flow criteria), although whether interventions to identify and treat these patients is feasible, improves outcomes, or is cost effective requires further investigation.

Our study has limitations. First, this is a post-hoc analysis, and the definition of CVC malfunction included blood flow as a criteria (a significant predictor of the malfunction outcome), which may lead to an overestimate of the true effect of blood flow as a predictor. Secondly, our protocol limited the number of sessions that could be performed with a CVC in the reversed position. At the time of our study, CVC reversal was believed to result in a reduction in dialysis adequacy. CVC reversal was the strongest risk factor in our univariate analysis, therefore future studies could prospectively evaluate this association over longer time periods. Finally, the generalizability of these results to other units where trisodium citrate is used as a locking solution may be limited.

## Conclusion

In conclusion, we found that HD session characteristics related to blood flow and blood processed were associated with an increased risk of CVC malfunction, while patient characteristics were not. Further study is needed to determine if the routine use of rt-PA as a locking solution in patients at increased risk of CVC malfunction will result in clinical and economic benefit.
